# Rationale and study design of the Adaptive study of IL-2 dose on regulatory T cells in type 1 diabetes (DILT1D): a non-randomised, open label, adaptive dose finding trial

**DOI:** 10.1136/bmjopen-2014-005559

**Published:** 2014-06-04

**Authors:** Frank Waldron-Lynch, Paula Kareclas, Kathryn Irons, Neil M Walker, Adrian Mander, Linda S Wicker, John A Todd, Simon Bond

**Affiliations:** 1 JDRF/Wellcome Trust Diabetes & Inflammation Laboratory, NIHR Cambridge Biomedical Research Centre, Cambridge Institute for Medical Research, University of Cambridge, Cambridge Biomedical Campus, Cambridge, UK; 2 Cambridge Clinical Trials Unit, Cambridge University Hospitals NHS Foundation Trust, Cambridge Biomedical Campus, Cambridge, UK; 3 MRC Biostatistics Unit Hub for Trials Methodology Research, Cambridge Institute of Public Health, Cambridge, UK

## Abstract

**Introduction:**

CD4 T regulatory cells (Tregs) are crucial for the maintenance of self-tolerance and are deficient in many common autoimmune diseases such as type 1 diabetes (T1D). Interleukin 2 (IL-2) plays a major role in the activation and function of Tregs and treatment with ultra-low dose (ULD) IL-2 could increase Treg function to potentially halt disease progression in T1D. However, prior to embarking on large phase II/III clinical trials it is critical to develop new strategies for determining the mechanism of action of ULD IL-2 in participants with T1D. In this mechanistic study we will combine a novel trial design with a clinical grade Treg assay to identify the best doses of ULD IL-2 to induce targeted increases in Tregs.

**Method and analysis:**

Adaptive study of IL-2 dose on regulatory T cells in type 1 diabetes (DILT1D) is a single centre non-randomised, single dose, open label, adaptive dose-finding trial. The primary objective of DILT1D is to identify the best doses of IL-2 to achieve a minimal or maximal Treg increase in participants with T1D (N=40). The design has an initial learning phase where pairs of participants are assigned to five preassigned doses followed by an interim analysis to determine the two Treg targets for the reminder of the trial. This will then be followed by an adaptive phase which is fully sequential with an interim analysis after each participant is observed to determine the choice of dose based on the optimality criterion to minimise the determinant of covariance of the estimated target doses. A dose determining committee will review all data available at the interim(s) and then provide decisions regarding the choice of dose to administer to subsequent participants.

**Ethics and dissemination:**

Ethical approval for the study was granted on 18 February 2013.

**Results:**

The results of this study will be reported through peer-reviewed journals, conference presentations and an internal organisational report.

**Trial registration numbers:**

NCT01827735, ISRCTN27852285, DRN767.

**Strengths and limitations of this study**

- This is an adaptive dose-finding trial that combines a new trial design with the use of immunological biomarkers to develop a new treatment for type 1 diabetes.
- The study incorporates detailed experimental medicine mechanistic studies that will investigate the actions of ultra-low dose IL-2 on the human immune system.
- The adaptive study design has required the development of new trial governance structures to allow data generated in the study to be rapidly analysed and utilised to inform dosing decisions.
- The study does not aim to determine the metabolic effects of treatment.

## Introduction

Type 1 diabetes (T1D) is the most common severe chronic autoimmune disease worldwide. The incidence of T1D is rising rapidly with a predicted increase in paediatric cases of 70% over the next 15 years in Europe.^1^ The aetiology of T1D is the autoimmune (loss of self-tolerance)-mediated destruction of insulin-producing pancreatic β cells leading to insulin deficiency and development of hyperglycaemia.^2^ At present, medical management of T1D focuses on intensive insulin replacement therapy to limit microvascular complications (retinopathy, nephropathy, neuropathy). Despite incremental improvement over the past 90 years clinical outcomes remain suboptimal with fewer than 5% of patients in the intensively treated group of the pivotal Diabetes Control and Complications Trial achieving glycaemic targets.^3^ The limiting factor for achieving euglycaemia was hypoglycaemia as a result of exogenous insulin treatment, that is, the tighter the glycaemic control the greater the frequency of hypoglycaemia.^4^ However, patients who had residual endogenous insulin function had a reduced level of microvascular complications and hypoglycaemia, which was most likely due to the preservation of the counter-regulatory responses to low blood sugars.^5^ These findings have led to intensive efforts to arrest the autoimmune process by novel immunotherapy and thereby preserve residual insulin production leading to improved clinical outcomes in T1D.

Genome-wide association studies have found that most genes contributing to T1D susceptibility encode proteins involved in immune regulation and immune function.^6^ In particular, several of the proteins are part of the interleukin 2 (IL-2) pathway that regulates T-cell activation and tolerance to self-antigens: IL-2, CD25, the α chain of the IL-2 receptor (*IL2RA*), *BACH2* and protein tyrosine phosphatase non-receptor type 2 (*PTPN2*).^7^ Phenotypic characterisation of CD25 expression on CD4 T-cell subsets has demonstrated that individuals carrying susceptibility alleles at *IL2RA* have memory CD4 T cells with reduced CD25 expression and less production of IL-2 on activation.^8^ Physiologically, IL-2 expression and signalling via the high-affinity trimeric IL-2 receptor is essential for the maintenance of self-tolerance and the prevention of autoimmunity.^9^

T regulatory cells (Tregs) and T effector (Teff) cells differ in their abilities to respond to IL-2 due to their distinct CD25 levels and the balance of their intracellular signalling molecules. In response to IL-2, Tregs intracellularly signal primarily via the pSTAT5 pathway while Teffs also activate the mitogen-activated protein kinase (MAPK) and PI3K/Akt pathways.^10^ ^11^ Importantly, Tregs have a greater sensitivity to IL-2 due to their higher expression of the high-affinity IL-2 receptor compared with Teff cells. Natural Killer (NK) cells also require higher concentrations of IL-2 to be activated since this subset primarily expresses the intermediate affinity IL-2 receptor that is composed of dimers of the β and γ chains.^12^ The higher sensitivity of Tregs for IL-2 opens a therapeutic window where ultra-low doses (ULD) of IL-2 therapy can be used to enhance Treg responses in patients with T1D without increasing Teff or NK cells.

Aldesleukin or proleukin is a human recombinant IL-2 product produced by recombinant DNA technology using a genetically engineered *Escherichia coli* strain expressing an analogue of the human IL-2 gene. The in vitro biological activities of the native non-recombinant compound have been reproduced with aldesleukin.^13^ Aldesleukin is produced by Prometheus Laboratories on behalf of Novartis Vaccine and Diagnostics.

High-dose aldesleukin is currently indicated for the treatment of adults with metastatic renal cell cancer (RCC)^14^ and metastatic melanoma skin cancer.^15^ Initial clinical trials in metastatic RCC administered intravenously 600 000 IU/kg of aldesleukin every 8 hours days 1–5 days followed by 9 days of rest and further treatment on days 15–19. In responders repeat cycles are administered in 12 week intervals up to a total of 3 cycles. Less than 10% of patients had a complete response to IL-2 therapy.^16^ Alternative regimens with subcutaneous aldesleukin have also been used. Aldesleukin is administered at 18×10^6^ IU every day for 5 days, followed by 2 days of rest. For the following 3 weeks 18×10^6^ IU is administered on days 1 and 2 of each week followed by 9×10^6^ IU on days 3–5. On days 6 and 7 no drug is administered. After 1 week's rest this 4-week cycle is repeated.^16^ The reduced dose regimens, although minimising side effects, yield substantially lower clinical responses than the high-dose protocol and are not considered effective treatment of metastatic renal cell carcinoma.^17^

In patients with HIV, clinical trials of aldesleukin therapy have been conducted to determine whether increasing the CD4 T cell count would improve clinical outcomes (opportunistic disease or death from any cause). The Subcutaneous Recombinant Interleukin-2 in Patients with HIV with Low CD4 Counts under Active Antiretroviral Therapy (SILCAAT) trial administered a dose of 4.5×10^6^ IU twice daily for 5 days for six cycles with each cycle 8 weeks apart. The Evaluation of Subcutaneous Proleukin in a Randomised International Trial (ESPRIT) delivered 7.5×10^6^ IU twice daily for 5 days for three cycles with each cycle 8 weeks apart. In both trials aldesleukin induced an increase in CD4 cell count as compared with antiretroviral therapy alone. However, no additional clinical benefit was observed in the aldesleukin plus antiretroviral therapy groups. Neither the SILCAAT nor ESPRIT trial included a mechanistic analysis so it is unclear whether the aldesleukin therapy induced a population of Tregs that may have blunted the Teff function.^18^

A combination phase 1 trial of rapamycin and aldesleukin in recently diagnosed patients with T1D has been reported. The rationale for this combination originated from murine studies where rapamycin and IL-2 had been shown to prevent diabetes but not to reverse it in the non-obese diabetic mouse model.^19^ Additional data from other murine models suggested that rapamycin selectively inhibits Teff function as compared with Treg function.^20^ Rapamycin was administered at 2 mg/day for 7 days followed by a dose adjustment to achieve a serum level of 5–10 ng/mL for 12 weeks. Aldesleukin was started concurrently and administered subcutaneously at 4.5×10^6^ IU once a day for 3 days for four cycles. The combination treatment resulted in a transient decrease in pancreatic β function (as measured by C-peptide decline) that resolved after discontinuation of rapamycin. As preservation of residual insulin production in the pancreas is critical to improved clinical outcomes, further studies in patients with T1D should avoid combining rapamycin and IL-2.^21^

Two recent successful trials of low-dose aldesleukin in graft versus host disease (GVHD) and hepatitis C virus (HCV) induced vasculitis (VASCU-IL2) have been reported. Patients with chronic GVHD who were resistant to glucorticoid therapy were treated with either 0.3×10^6^, 1×10^6^ or 3×10^6^ IU/m^2^/day of aldesleukin for 8 weeks. The numbers of CD4 Tregs increased in all patients without an increase in Teff cells. Patients had sustained clinical responses with extended therapy and this enabled tapering of glucorticoids.^22^ HCV vasculitis patients were treated with 1.5×10^6^ IU once a day for 5 days followed by 3×10^6^ IU for 5 days for three cycles on weeks 3, 6 and 9. The proportion of Treg cells increased during treatment without an increase in Teff cells. Increased natural killer (NK) cells and eosinophilia were also noted with aldesleukin treatment. Overall, patients with HCV vasculitis, an autoimmune condition, demonstrated clinical improvement on this regimen.^23^

There is substantial non-clinical, preclinical and clinical data supporting the possibility that IL-2 (aldesleukin) therapy can arrest the autoimmune-mediated destruction of pancreatic β cells by induction of functional Tregs that inhibit islet-specific autoreactive Teffs. However, prior to embarking on large proof-of-concept trials in T1D it is essential that the optimal doses of IL-2 that induce increases in Treg functions are determined while simultaneously defining the cellular outcomes of treatment by detailed immunophenotypic, genetic and epigenetic analysis of peripheral blood cell subsets from participants before, during and after IL-2 in order to define mechanisms and biomarkers.

## Methods

### Study design

**Figure 1:**
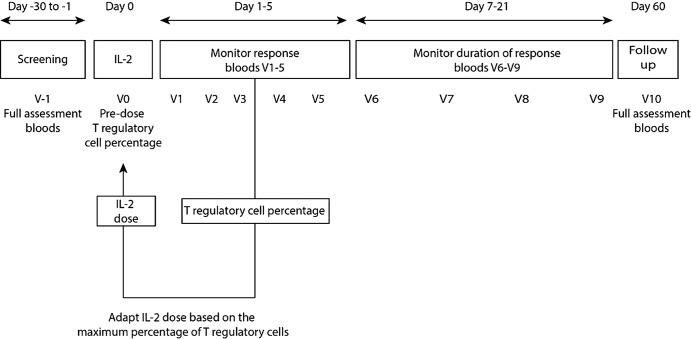
Study design for the adaptive phase of dose on regulatory T cells in type 1 diabetes. The primary endpoint of the study is the maximum percentage increase in Tregs from baseline over the first 7 days following treatment with ultra-low dose interleukin 2 (IL-2). The T regulatory data from all participants treated are then used to inform the IL-2 dose administered to subsequent participants thereby more efficiently accessing the dose–outcome relationship.

The DILT1D study is a 9-week, single centre non-randomised, single dose, open label, adaptive dose-finding trial. The study includes 12 visits: a screening visit, a treatment day, five visits to monitor the response to a dose of ULD IL-2, four visits to monitor the duration of response and a final follow-up visit on day 60 (figure 1). The DILT1D study has two phases: a learning phase and an adaptive phase. At the start of the study (learning phase) the first 10 participants will receive doses 0.04, 0.16, 0.6, 1, 1.5×10^6^ IU/m^2^ of IL-2, in ascending order with each of the doses being given to two patients before escalating the dose, and with at least a week between pairs of recruits. In the subsequent adaptive phase the data will be analysed sequentially after each participant is observed by fitting a candidate set of statistical models to the dose–response curve. Each model will provide an estimate and SE of the doses that achieve the two targets of a minimum Treg increase and a therapeutic Treg increase. Each model will also provide a recommended dose to assign to the next patient. The choice of doses will be approved by a dose determining committee (DDC) in the light of the reports and recommendations provided. The maximum dose of IL-2 that can be assigned is 1.5×10^6^ IU/m^2^. The study has been approved by Health Research Authority, National Research Ethics Service (13/EE/0020).

### Dose determining committee

The scope of the DDC is to review the interim analysis after the first 10 trial participants and then provide decisions regarding the choice of dose to administer to subsequent participants. The DDC will also review all safety data accumulated in the trial at each meeting. The DDC will be comprised of a statistician, a physician and a scientist drawn from the members of the Trial Management Committee or named in the trial delegation log. More than one member from each role (statistician, physician and scientist) can attend the meeting but each role is only allowed a single vote at the DDC meeting. A statistician, a clinician and a scientist are required to attend to reach a quorate. Decisions at the DDC meeting can be reached by a majority vote. The Trial Steering Committee can be called on by the chair to review any decisions that cannot be agreed on if requested to by other member(s) of the DDC. Given the safety role of the DDC, the chair is the chief investigator or if unavailable, the chief investigator may delegate the chair to another physician.

After the 10th participant has completed 7 days of follow-up after administration of the drug, data will be extracted from the trial database and analysed. The interim analysis will be performed by the members of the Trial Management Committee and will be delivered to the DDC within 10 working days of this date for review. Following the interim analysis, data will be extracted from the trial database after each participant has completed 7 days of follow-up. A report generated from the data will be delivered to the DDC within one to two working days of this date, the tight timelines enabling the next dose to be prescribed for a patient treated at the start of the following week.

The report generated from the data by the trial statistician for the DDC to review will include: plots of all the patient profiles (Treg response vs time); plots of the sequence of doses; a scatter plot of the primary endpoint (maximal percentage change of Treg, log-transformed) versus dose; the same scatter plot of the primary endpoint versus dose with superimposed fitted models with 95% confidence bands for a list of statistical models; estimated target doses and CIs; residual plots of each model fitted; raw output from statistical packages to double-check on convergence and finally a choice of dose decisions for future patients. The statistical models will initially include: linear, quadratic, cubic, Emax, Emax4 (four parameter) and logistic (four parameter).

### Study participants and recruitment

Potential participants will need to provide written informed consent before undergoing any trial-related procedures, including screening. Eligible participants will have a history of T1D with a duration of diabetes less than 24 months from diagnosis and be positive for at least one autoantibody (box 1). Participants will be excluded if they have a history or evidence of severe organ dysfunction, unstable T1D, pregnancy, malignancy, active autoimmune thyroid disease, active clinical infection, hepatitis B or C, HIV and/or organ transplantation (box 2).

Box 1 **Inclusion criteria**

Type 1 diabetes

18–50 years of age

Duration of diabetes less than 24 months from diagnosis

At least one positive autoantibody (anti-islet cell, anti-GAD, anti-IA2, anti-ZnT8)

Written informed consent

Box 2 **Exclusion criteria**

Hypersensitivity to aldesleukin or any of the excipients

History of severe cardiac disease

History of malignancy within the past 5 years (with the exception of localised carcinoma of the skin that had been resected for cure or cervical carcinoma in situ)

History or concurrent use of immunosuppressive agents or steroids

History of unstable diabetes with recurrent hypoglycaemia

Active autoimmune hyperthyroidism or hypothyroidism

Active clinical infection

Major pre-existing organ dysfunction or previous organ allograft

Women who are pregnant, lactating or intend to get pregnant during the study

Men who intend to father a pregnancy during the study

Donation of more than 500 mL of blood within 2 months prior to aldesleukin administration

Participation in a previous therapeutic clinical trial within 2 months prior to aldesleukin administration

Abnormal ECG

Abnormal full blood count, chronic renal failure (stages 3–5) and/or evidence impaired liver function

Positive HBsAg or HepC serology or HIV test

Any medical history or clinically relevant abnormality that is deemed by the principal investigator and/or medical monitor to make the patient ineligible for inclusion because of a safety concern

Potential participants can be informed of the study by several different systems depending on geographical location and participant preference. For local recruitment potential participants will be identified by their treating physicians, diabetes nurses and research nurses at Addenbrooke's Hospital or approved patient information sites. The contact details of identified potential participants, with their agreement, will be passed to the study team. For national recruitment participants who have registered with the ADDRESS-2 register^24^ or the D-GAP study^25^ will be contacted to determine whether they are interested in enrolling in the study. Details of the study will also be provided to patient groups and charities and will be posted on http://www.clinical-trials-type1-diabetes.com. There will also be a Facebook page and twitter feed for this study.

### The DILT1D study outcome measures

The primary endpoint is based on the percentage of CD4 Treg (defined as CD3CD4CD25^high^CD127^low^) cells within the CD3 CD4 T-cell gate following treatment with IL-2 as measured by fluorescence-activated cell sorting (FACS). The maximum value observed in each patient's profile over the first 7 days of the follow-up period will be identified and the percentage change from the baseline value defines the primary endpoint.

The following secondary outcomes will be measured following IL-2 treatment:

- Change in Treg number, phenotype and proliferation will be measured by FACS.
- Change in Treg cell epigenetic profile.
- Change in Teff number, proliferation and phenotype will be measured by FACS.
- Change in lymphocyte cell number, proliferation and phenotype subsets and NK and NKT cells will be measured by FACS and full blood count.
- Change in cytokines and soluble receptors.
- Change in metabolic control as measured by self-monitoring of blood glucose, laboratory measurement of blood glucose and glycated haemoglobin (HbA1c) and C-peptide.

The following exploratory endpoints will be measured:

- Change in intracellular T and NK cell signalling will be measured ex vivo by FACS following IL-2 treatment. An in vitro dose–response to IL-2 will also be performed to assess durable changes in intracellular T-cell signalling.
- Change in Treg function will be measured by T-cell suppression assay.
- Change in T cell, NK and peripheral blood mononuclear cell gene expression.
- Participants will be characterised for genotypes at T1D susceptibility genes related to the IL-2 pathway.

#### Safety assessments

Safety and tolerability assessments will include clinical history, insulin use, physical examination, temperature, blood pressure, heart rate, 12-lead ECGs, glucose, HbA1c, clinical laboratory tests and adverse event recording.

### FACS measurements and mechanistic analysis

The FACS for Treg (CD3, CD4, CD25, CD127) counts and proportions (%) that define the primary endpoint will be performed at the Department of Immunology, Addenbrooke's Hospital, Cambridge, a clinical laboratory that has been approved for good clinical practice. This assay will be carried out in a blinded fashion without the operators knowing the dose allocation for participants. The non-clinical mechanistic analysis for the secondary and exploratory endpoints for FACS immunophenotyping, Treg epigenetics, intracellular T and NK cell signalling, T-cell function genotype, gene expression analysis will be performed at the JDRF/Wellcome Trust Diabetes and Inflammation Laboratory, Cambridge Institute for Medical Research, University of Cambridge, Cambridge.

### Statistical methods

As an exploratory dose-finding study a formal sample size calculation is not appropriate. Simulation work shows that a sample size of 40 participants will give informative estimates of the target doses, assuming the underlying dose–response relationship can achieve the target responses within a safe range of doses and the between-patient variability does not dominate the dose–response relationship too much to be of practical clinical use.

A dose–response curve describing the relationship between the primary endpoint and the dose will be fitted for a selection of parametric models. Estimates and SEs for all parameters, including the interpatient variability, will be provided for all models, as well as an assessment of the goodness of fit for each model.^26^ An estimate, SE and 95% CIs will be produced for the doses associated with each of the different modelling assumptions that achieve the target response rates.

The target response rates are those that achieve a:

1. minimal Treg increase,
2. maximal Treg increase.

However, the numerical values that define these increases will only be defined in the light of the data provided by the initial 10 participants. After analysis by the DDC and following review by the TSC these targets will be fixed for the course of the trial.

Summary statistics of all endpoints measured at baseline will be produced. Continuous variables will report sample size, mean, SD, median, minimum and maximum. Categorical or binary variables will report sample size, counts and percentages.

All secondary and exploratory endpoints measured after treatment will be explored using graphical methods, such as scatter plots, to examine their relationship to dose and other explanatory endpoints measured at baseline. A regression framework will be used to quantify such relationships, allowing for adjustments for baseline covariates and time point; transformations of the response variable will be made where appropriate and allowances for correlations within participants and/or within related endpoints will be made.

## Discussion

Previous clinical trials involving the treatment of patients with newly diagnosed T1D with potential immunotherapeutics have embarked on large clinical proof-of-concept trials without first establishing the correct dose of the experimental agent in order to achieve the desired immunological outcome. Doses have been usually derived from experience of an agent in another disease entity such as in the case of teplizumab (non-Fc-binding anti-CD3) where the dose used in T1D is the same as that used in renal transplantation (OKT3).^27^ Similarly, the doses of rituximab (anti-CD20) and abatacept (CTLA-4Ig) when used to treat T1D have been derived from clinical experience in rheumatoid arthritis.^28–31^ In the case of otelixizumab (non-FcR-binding anti-CD3), experience from murine models was combined with limited human data to arrive at a dose. This has led, despite considerable efforts, to suboptimal outcomes in clinical trials of these agents, and in the case of otelixizumab, a complete failure in one trial due to a lack of therapeutic effect in humans.^32^

It is clear that new strategies need to be developed to rapidly determine the mechanisms of action of immunotherapeutic agents in patients with T1D prior to embarking on large phase II/III clinical trials.

The main goal of this adaptive mechanistic trial is to establish the best doses of IL-2 to administer in participants with T1D in order to:

- induce a minimal Treg increase,
- induce a maximal Treg increase.

Secondary goals are:

1. to determine the duration of Treg response from a single dose of IL-2,
2. to investigate the utility of biomarkers of IL-2 responsiveness in treated individuals.

Administration of a single ULD of IL-2 to participants with T1D will enable the determination of the response of the Treg population in this disease. By monitoring the Treg population over subsequent days and weeks we can determine the duration of the Treg population's increase in frequency and function and the return to baseline. It is essential that the optimal dose and duration of response of Tregs be established in T1D prior to the initiation of any future trials of IL-2. The dose and the frequency of dosing will determine whether aldesleukin is clinically acceptable for the long-term treatment of T1D. An empirically derived dose based on experience in other diseases may not be beneficial in T1D since the treatment protocols used in GVHD and HCV vasculitis gave a large rise in Treg population (eightfold and fourfold rise in Tregs from baseline, respectively) and alteration of Treg frequency is not a feature of T1D. Our aim in T1D will be to cause a small or physiological sustained increase in Treg frequency and function that may be maintained over the long term to induce tolerance to insulin-producing pancreatic β cells. This trial will provide the opportunity to determine the minimum dose of IL-2 that could be used to initiate treatment of patients with newly diagnosed T1D, and, in the future, to test the possibility that ULD IL-2 can prevent the onset of autoimmunity, which occurs many years before disease diagnosis. In addition, it will provide data regarding the duration of Treg response which can be used to estimate the frequency of IL-2 dosing in future trials.

An adaptive trial design is well suited to determine the dose response of Tregs to IL-2 therapy. Tregs are an appropriate biomarker since they are highly responsive to IL-2 therapy in humans at ULD and defects in their function are key to the development of T1D. By use of an adaptive design, following the learning phase of the trial where the first 10 start-up participants receive prespecified doses, Treg data from each participant treated can be used to inform the IL-2 dose administered to subsequent individuals in the trial thereby more efficiently accessing the dose–outcome relationship. In this manner Treg data from all participants enrolled will be used. Compared with a standard dosing trial an adaptive design has the advantage of not having to make definitive decisions prior to trial regarding dose and allocation to predesignated treatment groups.

By targeting the IL-2 pathway, one of the key aetiological pathways causing susceptibly to T1D, it will be possible to examine if IL-2 therapy rectifies known deficiencies by analysis of associated biomarkers. Individual participants will have their T-cell subsets followed longitudinally and characterised by deep immunophenotyping before and after treatment to determine the effects on CD25 and FOXP3 expression. Monitoring for increased proliferation (Ki-67) and the emergence of recent thymic emigrants (CD31 cells, increased T-cell receptor excision circles) will be performed. The stability of Tregs will be determined by phenotype (FOXP3, CTLA-4) and by epigenetic analysis of the regulatory regions of FOXP3 and genes associated with Treg function. Measurement of intracellular pSTAT5 signalling in lymphocytes will establish if qualitative defects in IL-2 signalling in T1D are corrected by therapy. Analysis of this panel of biomarkers may determine whether individual or combinations of assays may be useful in future trials to stratify participants with T1D on their ability to respond to IL-2 treatment.

## Supplementary Material

Author's manuscript
